# The influence of Konjac glucomannan on the functional and structural properties of wheat starch

**DOI:** 10.1002/fsn3.1598

**Published:** 2020-05-12

**Authors:** Ling Zhang, Lirong Zeng, Xuan Wang, Juncheng He, Qiong Wang

**Affiliations:** ^1^ College of Life Sciences and Health Wuhan University of Science and Technology Wuhan China

**Keywords:** crystalline structures, functional properties, Konjac glucomannan, leached amylose, starch hydrolysis rate, wheat starch

## Abstract

The aim of this study was to characterize the influence of Konjac glucomannan (KGM) on the functional and structural properties of wheat starch. Results showed that KGM significantly decreased the starch hydrolysis rate, with a lower level of rapidly digestible starch (RDS), and a higher content of slowly digestible starch (SDS). Besides, KGM decreased the content of leached amylose, while enhanced the swelling power, water‐holding capacity, freeze‐thaw stability, and paste clarity of wheat starch, which indicated a good improvement on the functional properties. Differential scanning calorimetry (DSC) and X‐ray diffraction patterns (XRD) manifested that the addition of KGM disrupted the original crystalline structures of wheat starch, which may result in the increased hydrolysis rate of starch. Interestingly, this did not consist with the decreased starch hydrolysis rate showed in the study. Moreover, FTIR results showed the existence of the interaction between KGM and starch. The morphological characterization demonstrated that the addition of KGM contributed to a more compact structure of freeze‐dried wheat starch. And KGM inhibited the expansion of starch granules and formed a barrier around the gelatinized starch. Therefore, the barrier around the starch granules and the interaction between KGM and starch were considered to be the important reasons that affected the starch digestibility.

## Graphical abstract

Konjac glucomannan decreased the starch hydrolysis rate.Konjac glucomannan changed the chemical structures of wheat starch.Konjac glucomannan formed a physical barrier around starch.

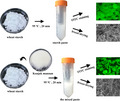

## 1 INTRODUCTION

Starch is the main nutrition components in the human diet, which widely exist in seed endosperm (Cornell & Eliasson, 2004). The physicochemical and nutritional properties of starch to a large extent determine the starchy food products quality (Maningat & Seib, 2010). Nowadays, various food additives (dietary fiber, polyphenol, enzymes, *etc.*) are used to improve the functional properties of starch (Han, Cheng, & Qiu, 2013; Matuda, Chevallier, de Alcântara Pessôa Filho, LeBail, & Tadini, 2008; Sun & Miao, 2019). Among these additives, dietary fiber can be used to modify the structural properties of starch, which intimately associated with the gelling and texture properties of starchy foods (Shevkani, Singh, Bajaj, & Kaur, 2017; Wüstenberg, 2015). Thus, adding dietary fiber to starchy food is a good choice for the functional food market.

Konjac glucomannan (KGM) is a kind of dietary fiber extracted from konjac tuber (*Amorphophallus konjac* K. Koch), which has been widely used as food additives in drinks and foods (Jimenez‐Colmenero, Cofrades, Herrero, Solas, & Ruiz‐Capillas, 2013; Silva, Ferreira, Bruschi, Britten, & Matumoto‐Pintro, 2016; Tatirat, Charoenrein, & Kerr, 2012; Xu, Liao, & Wang, 2012). Many researchers have investigated the influence of KGM on wheat‐based food products. A preliminary study showed that KGM addition could improve the overall quality of noodles made from low‐protein wheat flour through reinforcing the gluten network (Zhou et al., 2013). Further studies described that KGM could interact with gluten proteins via noncovalent interactions (Li, Zhu, Yadav, & Li, 2019; Wang et al., 2017). Moreover, KGM was also reported to affect the rheological and retrogradation properties of starch from different sources, which dependent on the structural compatibility and molecular interactions (Funami et al., 2005; Khanna & Tester, 2006; Yoshimura, Takaya, & Nishinari, 1998). Some researchers revealed the formation of strong hydrogen bonding between pea starch and konjac glucomannan (Chen, Liu, Chen, Chen, & Chang, 2008). However, there are few systematic reports related to the mechanism of KGM on the functional properties of wheat starch. The aim of this study was to characterize the influence of interaction between KGM and wheat starch on the functional and structural properties of wheat starch.

## 2 MATERIALS AND METHODS

### 2.1 Materials

Konjac glucomannan was purchased from Wuhan Yangyoudao health industry co., LTD company (Hubei, China). Starch from wheat, α‐amylase Type IX‐A from human saliva (activity 1,000–3,000 units/mg protein), pepsin from porcine gastric mucosa (activity 456 U/mg), amyloglucosidase from aspergillus niger (activity 30–60 units/mg protein), amylose from potato, and 3,5‐Dinitrosalicylic acid and fluorescein 5(6)‐isothiocyanate were purchased from Sigma‐Aldrich Co.

### 2.2 Measurement of in vitro digestibility

Starch digestibility was measured by Englyst, Kingman, and Cummings (1992) with some modifications. Wheat starch 0.2 g was gelatinized in 2 ml distilled water together with KGM (0%, 5%, 10%, and 15%) at 95°C for 20 min. After cooled to room temperature, the starch paste sample was immersed in 10 ml pepsin solution (456 U/mg/100 ml 0.01 M KCl‐HCl buffer, pH 1.2) with continuously oscillated at 37 ℃ for 60 min and volumetric to 25 ml with 0.1 M sodium phosphate buffer (pH = 7.5). Next, the samples were added with 5 ml α‐amylase solution (2 U/mL) and incubated at 37°C for 120 min. Then, 1 ml of the reaction solution was obtained from the mixture system every ten minutes. When the enzyme activity was rapidly inactivated at 95°C for 5 min, 3 ml 0.2 M sodium acetate buffer (pH = 4.75) and 21 μl amyloglucosidase solution (40 U/ml) were added to each removed reaction solution and incubated at 60°C for 50 min. Determination of sugar was obtained by the colorimetric method with 3,5‐Dinitrosalicylic acid. The optical density (OD value) was read at 520 nm, and the content was calculated by the calibration curve plotted for glucose. Starch was calculated as glucose × 0.9. According to the different hydrolysis percentage at each time point, the values for the different levels of starch fractions (RDS, SDS, and RS) were calculated using the following calculations:

$$\text{RDS}\left(\%\right)=G20\times 0.9/\text{TS}$$

$$\text{SDS}\left(\%\right)=\left(G120-G20\right)\times 0.9/\text{TS}$$

$$\text{RS}\left(\%\right)=\left[TS-\left(\text{RDS}+\text{SDS}\right)\right]/\text{TS}$$

where RDS‐rapidly digestible starch, SDS‐slowly digestible starch, RS‐resistant starch, TS‐the total starch content, G20‐starch hydrolysis at 20 min, and G120‐starch hydrolysis at 120 min.

### 2.3 Leached amylose

Wheat starch 0.1 g together with KGM was added to 1 ml 95% ethanol solution and 9 ml 1 mol/L NaOH solution and gelatinized at 95°C for 10 min, and then, 0.5 ml 1 mol/L acetic acid solution was added to lower the pH (pH ≈ 5.5). The content of amylose leached during gelatinization was obtained by the iodine colorimetry with I_2_­KI solution. The standard curve was made with amylose as a standard substance (Duan, Donner, Liu, Smith, & Ravenelle, 2012).

### 2.4 Swelling measurement

The swelling test was determined based on a reported method (Lu, Brennan, Serventi, Mason, & Brennan, 2016) with slight modifications. Wheat starch 0.2 g together with KGM (0%, 5%, 10%, and 15%) was immersed in 10 ml deionized water in a centrifuge tube, which was heated at 95 ℃ for 20 min. Then, samples were centrifuged at 3,800 g for 25 min; the sediment was dried and weighed. Swelling ratio was calculated from the weight of sediment divided by the dry weight of samples.

### 2.5 Water absorption capacity (WAC) measurement

Determination of water absorption capacity (WAC) of starch was according to Nawab, Alam, and Hasnain (2014). About 0.4 g starch sample together with KGM (0%, 5%, 10%, and 15%) was immersed in 10 ml distilled water. After left at room temperature for 30 min, the sample was centrifuged at 4,000 g for 15 min. The wet weight of the starch sample was weighed and dried under standardized conditions. Water absorption (WAC) was calculated as follows:

$$\text{WAC}\left(g/g\right)=\left(M_{m}-m\right)/m\times 100.$$

where, M_m—_the weight of wet sediment [g]; M—the dry weight of samples [g].

### 2.6 Freeze‐thaw stability (FTS) measurement

Freeze‐thaw stability was measured using Wattanachant’ method (Wattanachant, Muhammad, Hashim, & Rahman, 2003). Wheat starch 0.3 g together with KGM (0%, 5%, 10%, and 15%) was immersed in 10 ml deionized water and heated and then froze at −20 ℃ for 24 hr and thawed at room temperature referred as a freeze‐thaw cycle. The thawed sample was centrifuged at 3,800 g for 20 min. The supernatant was eliminated from the sample, then which was freeze and thawed repeatedly for four times. The freeze‐thaw stability was represented as water loss and calculated by the following formula:

$$\text{Water loss}\left(\%\right)=\left(m_{m}-m\right)/m_{m}\times 100.$$

where, m_m—_The weight of starch paste[g]; m—the weight of starch paste precipitation[g].

### 2.7 Paste clarity measurement

The paste clarity of wheat starch was measured concerning to Waterschoot, Gomand, Fierens, and Delcour (2015). Wheat starch 0.1 g together with KGM (0%, 5%, 10%, and 15%) was immersed in 10 ml deionized water and then stored at refrigerated temperature for a week. The absorbance of the starch samples was read at 650 nm. The conversion relationship between absorbance and transmittance (T) was as follows:

$$\text{Absorbance}\left(A\right)=2-\log\left(\%T\right).$$

$$\text{Transparency}\left(\%T\right)\;=10^{2-A}$$

### 2.8 Differential scanning calorimetry (DSC)

Wheat starch 5 mg together with KGM (0%, 5%, 10%, and 15%) was dissolved and mixed with distilled water in a preweighted aluminum pot and placed at 4°C for 12 hr until the water was completely in equilibrium with the sample. The condition was set to 10°C/min speed heat from 20 ℃ to 120 ℃ in DSC instrument, as a comparison with an empty plate.

### 2.9 X‐ray diffraction (XRD)

The diffraction patterns of freeze‐dried starch samples were determined by X‐ray diffractometer (X'Pert Pro, PANalytical B.V., Almelo, Netherlands) running at 40 kV and 40 mA. Samples were scanned in the range from 10 to 55° (2θ) (Chen, Ren, Zhang, Tong, & Rashed, 2015). The relative crystallinity of starch samples through the analysis of Software JADE 5.0 is calculated from the area of the crystalline peak divided by amounts of all the area of peak (the amorphous and crystalline peak).

### 2.10 Fourier transform infrared spectroscopy (FTIR)

Wheat starch 5 g together with KGM (0%, 5%, 10%, and 15%) was dissolved in 40 ml of deionized water and stir well and then pasted on 95°C for 20 min. After cooling to room temperature, freeze‐dried starch samples were obtained by vacuum freeze‐drying and pulverized through 100 mesh sieve. The FTIR spectra of four freeze‐dried starch samples were tested at 4,000 ~ 400 cm^−1^ band. The resulting data were obtained in 64 scans at a resolution of 4 cm^−1^.

### 2.11 Particle size analysis

Particle size analysis of freeze‐dried wheat starch samples was evaluated by Malvern laser particle size analyzer (Mastersizer 3,000). The results were expressed in terms of the volume distributions of freeze‐dried wheat starch. The mean particle size parameters were reported as D[4,3] (volume‐weighted mean) and D[3,2] (surface‐weighted mean). Each sample was performed in three replicates.

### 2.12 Scanning electron microscopy (SEM)

Freeze‐dried wheat starch samples were cut into cubes, and morphological properties of starch/ KGM samples were obtained by a scanning electron microscopy (Sirion 200, FEI, Netherlands) at a voltage of 15 kV. The starch sample was adhered to double‐sided coated carbon tape and fixed on the aluminum frame and then coated with gold–palladium. A meaningful freeze‐dried starch sample images were obtained at 1,200 × magnification.

### 2.13 Confocal light scanning microscopy (CLSM)

The internal structure of starch granules was investigated by using confocal laser scanning microscopy (CLSM) (Funami et al., 2008). Native wheat starch and mixed samples of wheat starch (5%, 10%, 15% KGM based on the mass of starch) were dissolved in distilled water and heated at 95°C for 20 min to produce gelatinized starch samples. Starch samples were stained at room temperature overnight using 20 μL 2 mg/ml FITC. The samples were observed by using a confocal laser electron microscopy (FLUOVIEW FV3000, OLYMPUS, Japan) under a fluorescence mode by a × 60 oil‐immersed objective lens. A small sample was dropped onto the glass slide. Excitation and emission for FITC were at 488 nm and 525 nm, respectively.

### 2.14 Statistic analysis

Data in our study were presented as means ± *SEM* of triplicate. Analysis of variance was used to contrast the significant difference by using one‐way analysis of variance (ANOVA). The significant differences (*p < .05*) were evaluated and displayed by different letters.

## 3 RESULTS AND DISCUSSION

### 3.1 In vitro digestibility

**FIGURE 1:**
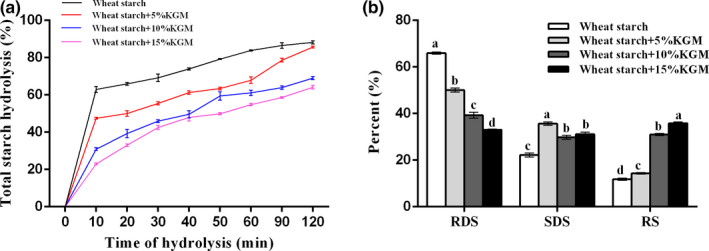
Effects of Konjac glucomannan on starch in vitro digestion. a, The hydrolysis percentage of wheat starch samples. b, The content of RDS, SDS, and RS in wheat starch samples. KGM‐Konjac glucomannan; RDS‐rapidly digestible starch; SDS‐slowly digestible starch; RS‐resistant starch. Data are given as mean ± *SEM*, calculated from three replicates. Values within the column followed by different letters indicate significant difference (*p* < .05)

The effects of Konjac glucomannan (KGM) on starch in vitro digestion were investigated. As shown in the Figure 1a, the hydrolysis percentage of native starch at 10 and 120 min were 62.98% and 88.19%, respectively. With the addition of KGM, there was a significant decrease in the rate of starch digestibility. Especially, the mixed sample containing 15% KGM had the most significant reduction at 10 min and 120 min, which the hydrolysis percentages were 22.7% and 64%, respectively. This result was supported by the previous study that the starch digestibility was affected by the presence of apple dietary fibers, inulin, guar gum, and xanthan gum (Bae, Jun, Lee, & Lee, 2016; Brennan, Kuri, & Tudorica, 2004; Roberts, 2011).

The addition of KGM had a positive effect on the content of rapidly digestible starch (RDS), slowly digestible starch (SDS), and resistant starch (RS). As shown in the Figure 1b, the content of RDS was decreased from 65.93% to 33.03% for wheat starch enriched with KGM, while the content of SDS was increased from 22.26% to 31.12%. Also, an increase in RS ranged from 11.81% to 35.85% was observed (Figure 1b). It was worth noting that the addition of 5% KGM showed the highest SDS content. This can be explained by the changed content of digestible starch, which consists of RDS and SDS. Compared with 5% KGM, the content of RS increased more significantly after the addition of 10% and 15% KGM. This result indicated that the content of digestible starch in the addition of 5% KGM was significantly higher than that of 10% and 15% KGM. As the RDS content was decreased regularly by the addition of KGM, it is reasonable that the addition of 5% KGM showed the highest SDS content. Thus, the addition of KGM was beneficial to prevent rapid degradation of starch and reduced postprandial blood sugar.

### 3.2 Effects of KGM on leached amylose ratio, swelling power, and water‐holding capacity of wheat starch

**FIGURE 2:**
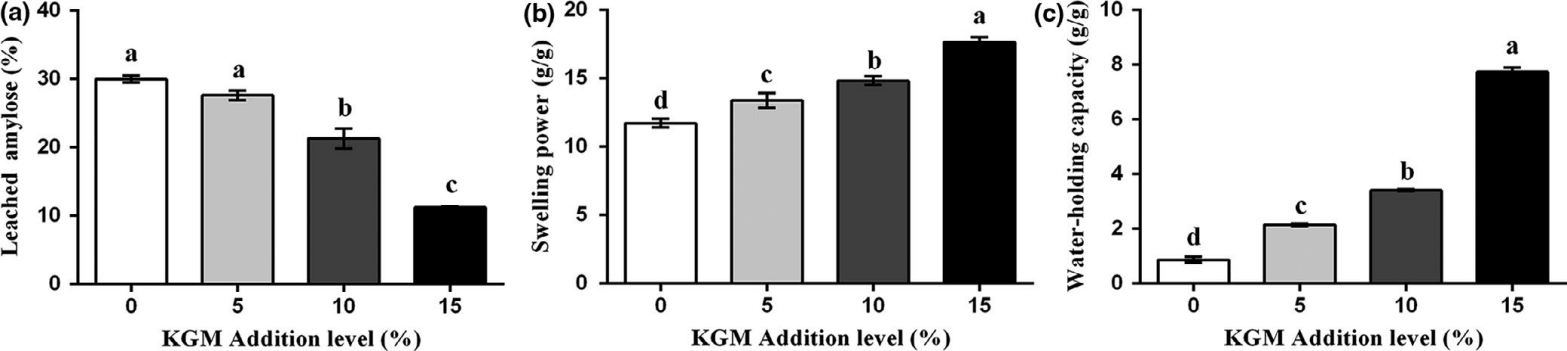
Effects of Konjac glucomannan on (a) leached amylose ratio (b) swelling power and (c) water absorption capacity of wheat starch samples. Data are given as mean ± *SEM*, calculated from three replicates. Values within the column followed by different letters indicate significant difference (*p *< .05)

As shown in the Figure 2a, the leached amylose ratio of native wheat starch and the mixed samples were 30%, 27.62%, 21.31%, and 11.23%, respectively. Compared with the control sample, the addition of 10% and 15% KGM significantly decreased the leached amylose ratio, while the addition of 5% KGM showed no significant influence. During gelatinization processing, the orderly arrangement of amylose and amylopectin in wheat starch granules was destroyed. The amylose that leached out of the starch granules is similar to the KGM in structure, which makes it could dissolve in the hydrophilic colloid (Chen et al., 2008). The lower level of leached amylose suggested that the KGM may have formed a barrier around the starch that preventing the diffusion of amylose during the gelatinization (Funami et al., 2005). Besides, the barrier around the starch granules reduced the area of contact between starch and enzymes, causing the increasing of RS content (Dupuis, Liu, & Yada, 2014). Moreover, the leached amylose would interact with KGM that led to the formation of KGM–amylose complex, which might generate more SDS (Nawab et al., 2014).

Compared with native wheat starch, the addition of KGM significantly improved the swelling power from 11.73 to 17.66 g/g (Figure 2b), and the water‐holding capacity increased from 0.86 to 7.73 g/g (Figure 2c). This did not consist of the results of previous studies that hydrocolloids inhibited the swelling and starch gelatinization (Chen et al., 2017; Zhang et al., 2018). The increased swelling power and water‐holding capacity of the KGM–starch mixtures may be due to the structural properties of the KGM. Konjac glucomannan is a nonionic polysaccharide hydrophilic colloid, consisting mainly of hydroxyl groups and the acetyl groups (Silva et al., 2016). Due to the hydroxyl groups, KGM competed with starch for water molecules, which inhibited the expansion of wheat starch. On the other hand, hydrated KGM molecules expanded rapidly and could cause a change in conformation, thereby resulting in the increased swelling power and water‐holding capacity of the KGM–starch mixtures.

### 3.3 Effects of KGM on freeze‐thaw stability and paste clarity of wheat starch

**FIGURE 3:**
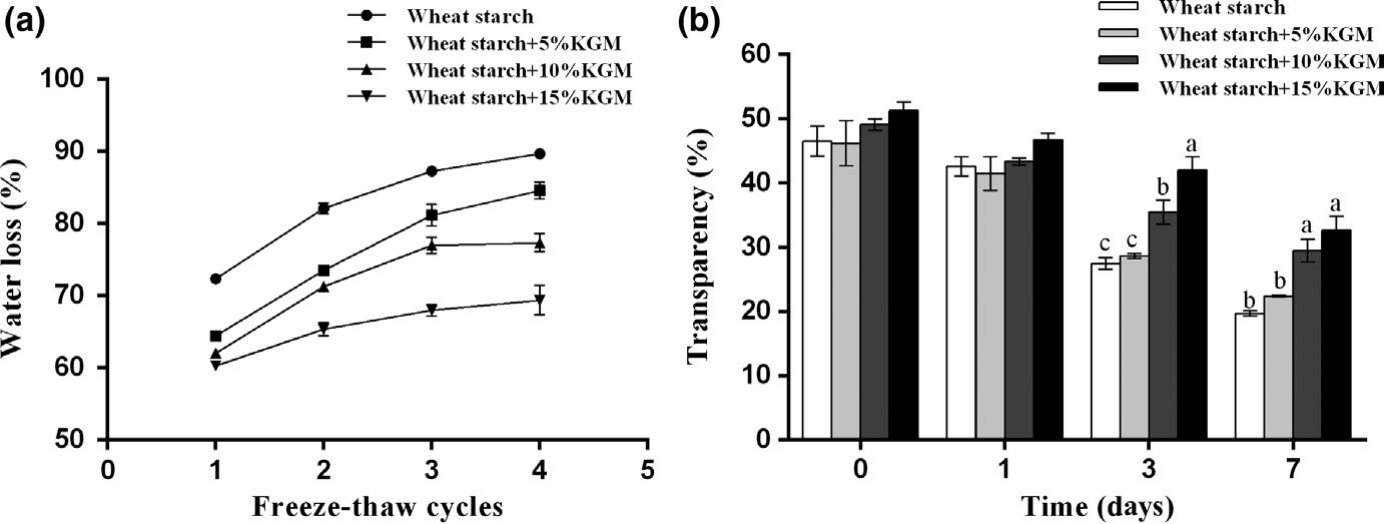
Effects of Konjac glucomannan on (a) freeze‐thaw stability and (b) paste clarity of wheat starch samples. Data are given as mean ± *SEM*, calculated from three replicates. Values within the column followed by different letters indicate significant difference (*p *< .05)

Freeze‐thaw stability is detected to understand the ability of frozen wheat starch with KGM addition to retaining their shape and texture after thawing. As shown in the Figure 3a, the water loss after the first cycle was 72.35% and increased to 89.68% after the fourth freeze‐thaw cycle. The water loss of 5%, 10%, and 15% Konjac glucomannan/starch mixed samples after the first freeze‐thaw cycle was 64.44%, 62.02%, and 60.26%, while after the fourth freeze‐thaw cycle, the water loss was 84.55%, 77.33%, and 69.35%, respectively. The addition of KGM significantly reduced water loss during the freeze‐thaw process. A lower water loss means better water retention and a lower rate of retrogradation (Ye et al., 2018). This result indicated that KGM could inhibit the retrogradation of wheat starch gel and enhance freeze‐thaw stability.

Paste clarity determines the appearance and use of starch products (Yadav, Kumar, & Yadav, 2016). As shown in Figure 3b, the transmittance of wheat starch decreased gradually with the extension of time. In a short period (1st), there was no significant change in transparency among the samples. From the 3rd day to the 7th day of the experiment, the transmittance was significantly increased in wheat starch added with 10% and 15% KGM. The increased transmittance indicated the less retrogradation of wheat starch (Fukuzawa, Ogawa, Nakagawa, & Adachi, 2016). Also, this result was supported by the reduced amylose which detected above, as lower amylose starches are easily dispersed (Singh, Kaur, Sandhu, Kaur, & Nishinari, 2006; Yamamori, 2009).

### 3.4 Effects of KGM on the chemical structure of wheat starch

**FIGURE 4:**
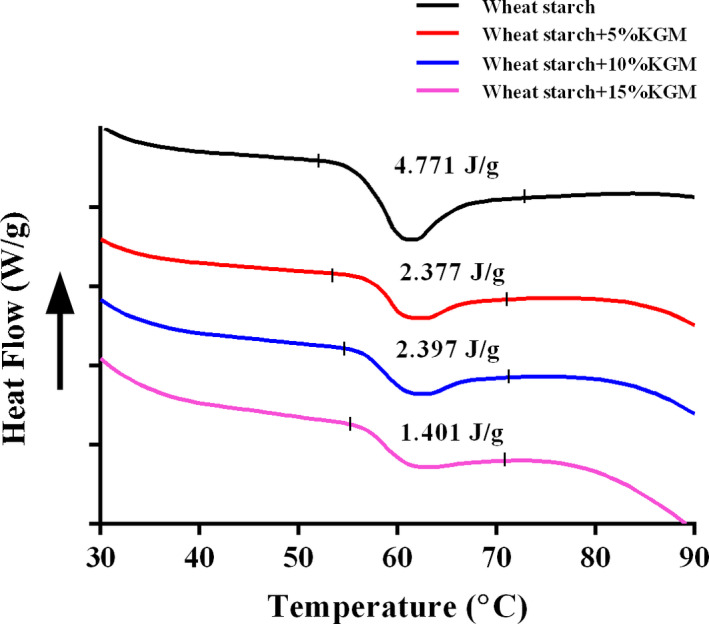
Effects of Konjac glucomannan on thermal properties of wheat starch samples

As can be seen in the Figure 4, all starch samples had only one endothermic peak (a single Tp), indicating the good compatibility between KGM and wheat starch. The onset temperature (To) and the endothermic peak (Tp) were not significantly affected by the addition of KGM, while the gelatinization enthalpy (ΔH) decreased from 4.771 to 1.401 J/g, respectively. The enthalpy change (ΔH) was a reflection of the formation of ordered structures (Wang, Li, Copeland, Niu, & Wang, 2015). Thus, the decrease in gelatinization enthalpy (ΔH) demonstrated the disruption of crystalline structures on wheat starch caused by KGM (Singh, Singh, Kaur, Sodhi, & Gill, 2003; Wang, et al., 2017). The effect on the gelatinization enthalpy (ΔH) was not consistent with previous studies of polysaccharides (e.g., HPMC, CMC, xanthan gum, apple pectin, tea polysaccharide) (Singh, Singh, Isono, & Noda, 2010; Zhou, Wang, Zhang, Du, & Zhou, 2008). This may be due to the different polysaccharide structure and physical properties (Funami et al., 2005).

**FIGURE 5:**
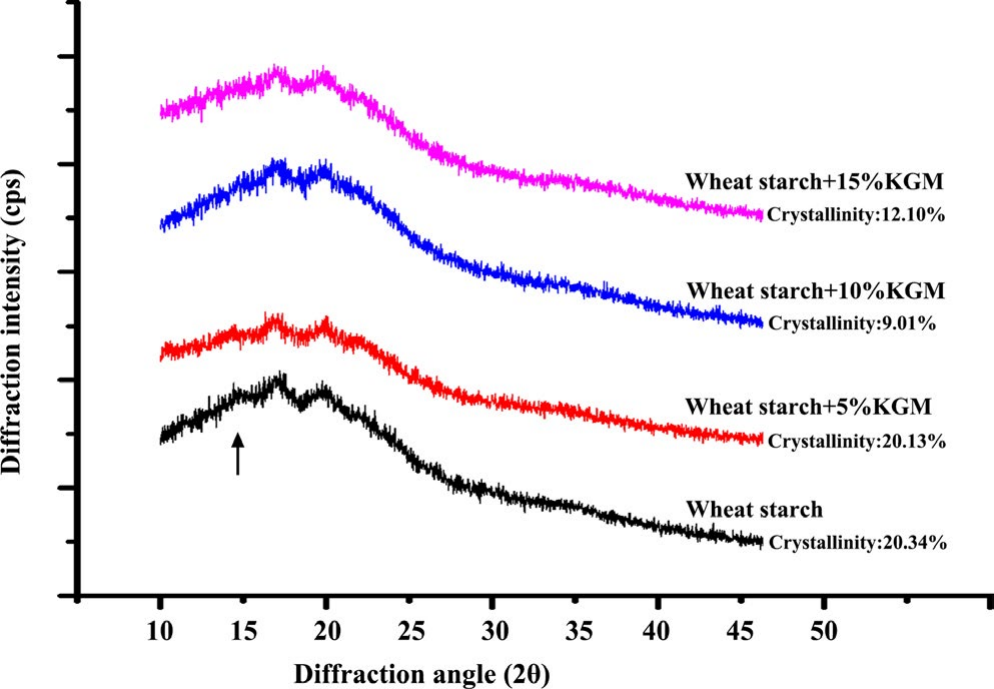
X‐ray diffractometry of freeze‐dried wheat starch samples. The arrow indicates the gradually disappeared crystalline diffraction peak at 15°

Four obvious crystalline diffraction peaks at 2θ close to 15°, 17°, 18°, and 22° were showed in the wheat starch system, which displayed a typical A‐type XRD crystal structure (Figure 5). With the addition of KGM, the crystalline diffraction peaks at 15° disappeared gradually, and the relative crystallinity values of wheat starch containing different concentrations of KGM were 20.34%, 20.13%, 9.01%, and 12.10%, respectively. The decreased crystallinity values indicated that the addition of KGM destroyed the original crystalline domains of wheat starch (Chen et al., 2008). This result was supported by the DSC analysis showed above. The crystallite disruption could facilitate rapid enzymatic hydrolysis (Zhang et al., 2018). Thus, the decreased crystallinity domains caused by Konjac glucomannan may increase the hydrolysis rate of starch. Interestingly, this did not consist with the decreased starch hydrolysis rate showed in the study. Therefore, the barrier around the starch granules and the interaction between KGM and amylose were confirmed to be the important reasons that influenced the starch digestibility, which discussed in the leached amylose experiments.

**FIGURE 6:**
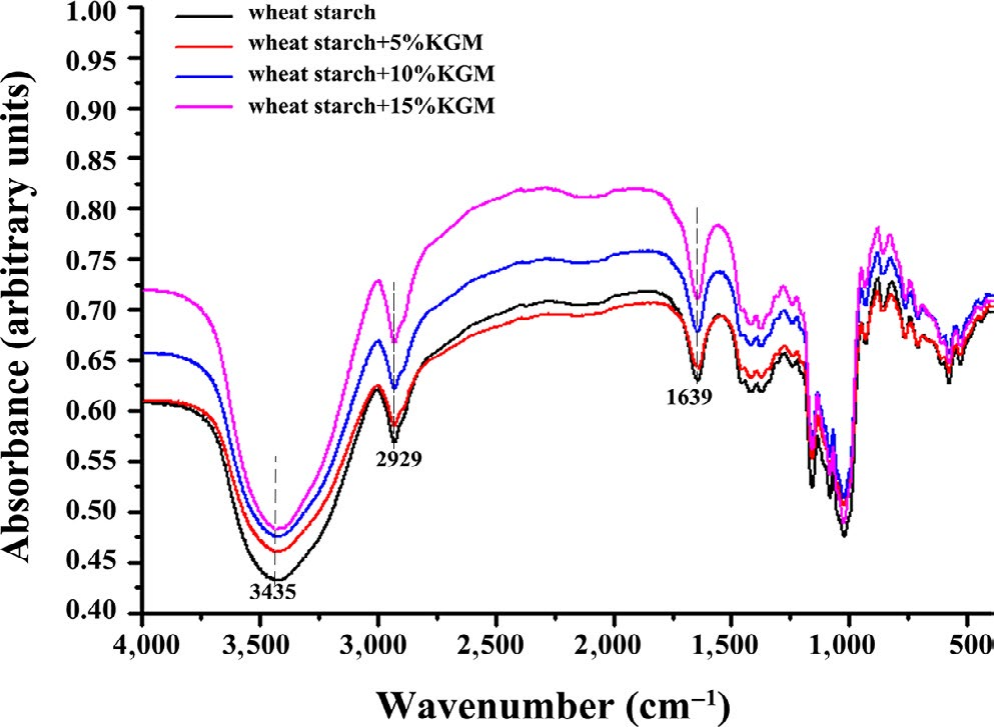
Fourier transform infrared spectroscopy of freeze‐dried wheat starch samples

To evaluate the interaction between KGM and wheat starch, the functional groups of the native wheat starch and the mixture were analyzed (Figure 6). It can be seen that the adsorption peaks at 1639 cm^−1^, which attributed to the C = O groups, were increased with the addition of KGM, confirming the good compatibility between KGM and wheat starch (Li, Li, Geng, Song, & Wu, 2017). Also, the absorption peaks at approximately 3,435 and 2,929 cm^−1^, which attributed to the vibration of O‐H stretching and the C‐H deformation of the glucose unit, were significantly enhanced with the addition of KGM. This phenomenon demonstrated the occurrence of the hydrogen bond between KGM and starch (Yuan, Wang, & Mu, 2018).

### 3.5 Effects of KGM on the average particle diameter

**FIGURE 7:**
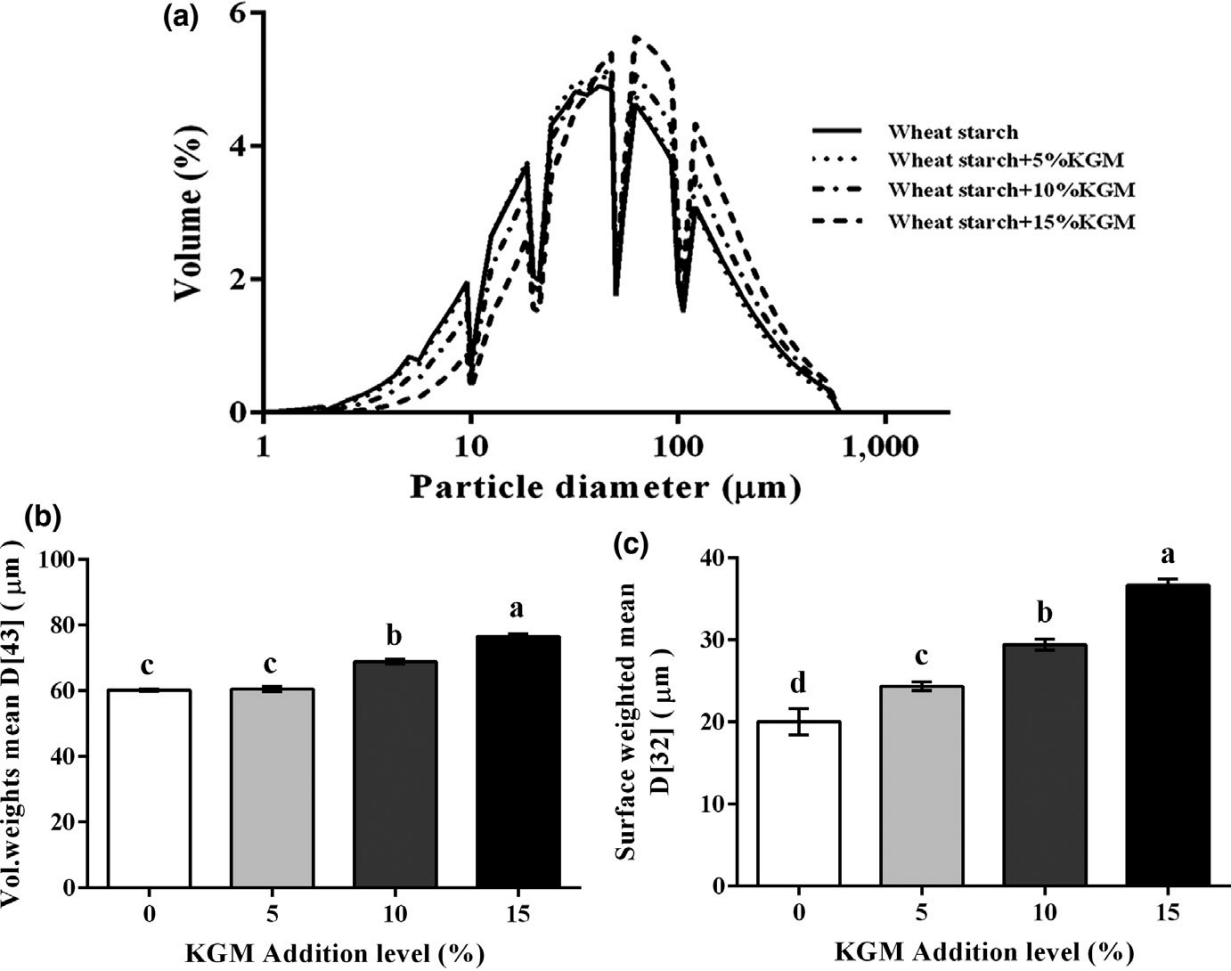
Average particle distributions. (a) The volume distributions of freeze‐dried wheat starch. (b) Volume‐weighted mean D[43] of freeze‐dried wheat starch. (c) Surface‐weighted mean D[32] of freeze‐dried wheat starch. Data of mean particle size D[43] and D[32] are shown as mean ± *SEM*. Values of D[43] and D[32] within the column followed by different letters indicate significant difference (*p* < .05)

For all starch samples, KGM addition led to an increased content of large particles of freeze‐dried wheat starch (Figure 7a). The surface‐weighted mean diameter D[3,2] of all starch samples was 20.05, 24.37, 29.46, and 36.69 μm, while the volume‐weighted mean diameter D[4,3] of all starch samples was 60.32, 60.51, 68.88, and 76.63 μm, respectively (Figure 7b, c). As we can see from Figure 7c, the addition of 10% and 15% KGM significantly increased the droplet size D[4,3], while the addition of 5% KGM showed no significant increase. This trend was in agreement with the changes in the leached amylose ratio and paste clarity (Figures 2a and 3b). Thus, the increase in the large particles of freeze‐dried wheat starch may be due to the improved gelatinizing structure of starch, which caused by the interaction between KGM and starch molecules.

### 3.6 Effects of KGM on the morphological characterization of wheat starch

**FIGURE 8:**
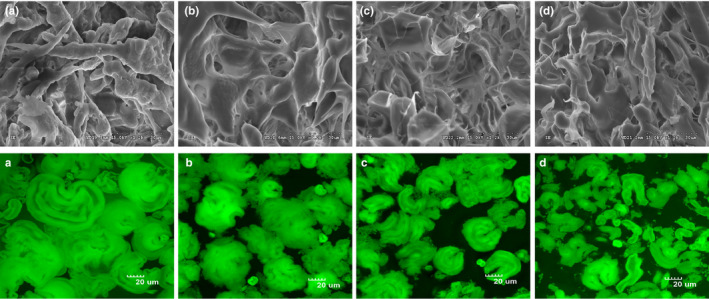
Effects of Konjac glucomannan on microstructure of wheat starch samples. *SEM* images of freeze‐dried wheat starch samples with (a) Native, (b) 5% Konjac glucomannan, (c) 10% Konjac glucomannan, and (d) 15% Konjac glucomannan. CLSM images of gelatinization wheat starch samples with (A) Native, (B) 5% Konjac glucomannan, (C) 10% Konjac glucomannan, and (D) 15% Konjac glucomannan

The morphological characterization of freeze‐dried wheat starch samples found that all the starch granules had been broken and a gel network structure was formed after gelatinization (Figure 8a–d). A more regular and lamellar structure was clearly shown on the surface of wheat starch with KGM addition of 5%, 10%, and 15% compared with the native wheat starch. The results suggested that KGM interacted with starch and contributed to a more compact starch structure.

Further morphological characterization of gelatinized starch was performed through confocal laser scanning microscopy (CLSM). During gelatinization, starch granules persist as highly swollen fragile forms, commonly termed granule “ghosts” (Debet & Gidley, 2007). The swollen starch granules were observed on the CLSM images. As can be seen, the starch ghost gradually decreased with the addition of KGM, while natural wheat starch expanded significantly after gelatinization (Figure 8). This result showed that KGM competed with starch for water molecules, inhibited the expansion of starch granules, and formed a barrier around starch to reduce the area of contact between starch and digestive enzymes.

## 4 CONCLUSION

Konjac glucomannan significantly decreased the starch hydrolysis rate, reduced the content of RDS, and increased the content of SDS and RS and thus slowed down the release of glucose from starch and reduced postprandial blood sugar. The lower level of leached amylose suggested that the KGM may have formed a barrier around the starch, which prevented the diffusion of amylose during the gelatinization. And the leached amylose would interact with KGM that led to the formation of KGM–amylose complex. Due to the strong hydrophilic ability, KGM increased the swelling power and water‐holding capacity of the KGM–starch mixtures. Besides, KGM was identified to enhanced freeze‐thaw stability and paste clarity of wheat starch. All these findings demonstrated that KGM improved the functional properties of starch. It was confirmed by the results of the chemical structures and morphologies characterization, the barrier around the starch granules and the interaction between the leached amylose and KGM were responsible for the reduced starch digestibility.

## CONFLICT OF INTEREST

There is none potential conflict between authors and others that bias our work.

## ETHICAL APPROVAL

This study does not involve any human or animal testing.
